# Circulating T lymphocyte subsets, cytokines, and immune checkpoint inhibitors in patients with bipolar II or major depression: a preliminary study

**DOI:** 10.1038/srep40530

**Published:** 2017-01-11

**Authors:** Wei Wu, Ya-li Zheng, Li-ping Tian, Jian-bo Lai, Chan-chan Hu, Peng Zhang, Jing-kai Chen, Jian-bo Hu, Man-li Huang, Ning Wei, Wei-juan Xu, Wei-hua Zhou, Shao-jia Lu, Jing Lu, Hong-li Qi, Dan-dan Wang, Xiao-yi Zhou, Jin-feng Duan, Yi Xu, Shao-hua Hu

**Affiliations:** 1 Collaborative Innovation Center for Diagnosis and Treatment of Infectious Diseases, State Key Laboratory of Diagnostic and Treatment of Infectious Diseases, the First Affiliated Hospital, Zhejiang University School of Medicine, Hangzhou 310003, China; 2 Zhejiang University School of Medicine, Hangzhou 310058, China; 3 Mental Health Center, Zhejiang Xiaoshan Hospital, Hangzhou 311200, China; 4 Department of Psychiatry, Shaoxing Seventh Hospital, Shaoxing 312000, China; 5 Department of Psychiatry, the First Affiliated Hospital, Zhejiang University School of Medicine, Hangzhou 310003, China; 6 The Key Laboratory of Mental Disorder Management in Zhejiang Province, Hangzhou 310003, China

## Abstract

This study aimed to investigate the less known activation pattern of T lymphocyte populations and immune checkpoint inhibitors on immunocytes in patients with bipolar II disorder depression (BD) or major depression (MD). A total of 23 patients with BD, 22 patients with MD, and 20 healthy controls (HCs) were recruited. The blood cell count of T lymphocyte subsets and the plasma level of cytokines (IL-2, IL-4, IL-6, IL-10, TNF-α, and IFN-γ) were selectively investigated. The expression of T-cell immunoglobulin and mucin-domain containing-3 (TIM-3), programmed cell death protein 1 (PD-1) and its ligands, PD-L1 and PD-L2, on T lymphocytes and monocytes, was detected. In results, blood proportion of cytotoxic T cells significantly decreased in BD patients than in either MD patients or HCs. The plasma level of IL-6 increased in patients with BD and MD. The expression of TIM-3 on cytotoxic T cells significantly increased, whereas the expression of PD-L2 on monocytes significantly decreased in patients with BD than in HCs. These findings extended our knowledge of the immune dysfunction in patients with affective disorders.

Mounting evidence of immune imbalance in affective disorders has sprung up in recent decades, pointing to a role of inflammatory burden in affected patients^1^,^2^. The crosstalk between inflammatory abnormalities and affective disorders has been extensively investigated from various perspectives, including cytokines, chemokines, antibody titers and immune cell numbers^3^,^4^,^5^,^6^,^7^. Albeit remarkably diverse, consistent findings exist on the blood level change of several cytokines, especially interleukin-6 (IL-6) and tumor necrosis factor-α (TNF-α), during the acute or chronic phase of major depression (MD) and bipolar II disorder depression (BD)^8^,^9^. Data regarding the activation pattern of immune cells in affective disorders are limited^10^, and the macrophage hypothesis of depression remains controversial^11^. Some studies demonstrated decreased proportion of monocytes (CD14+), whereas others reported increased frequency of monocytes in patients with BD^12^,^13^. Besides, reduced proportion of cytotoxic T cells (CD3+CD8+) has also been presented in patients with BD^13^. Of note, little attention has been focused on the role of immune checkpoint inhibitors in mediating the interplay between immune dysfunction and affective disorders. T-cell immunoglobulin and mucin-domain containing-3 (TIM-3), known as an inhibitory regulator molecule, may regulate macrophage activation and cytokine production^14^. Programmed cell death protein 1 (PD-1) also functions as an inhibitory immune checkpoint and negatively regulates immune response^15^,^16^. Moreover, TIM-3 and PD-1 both may weaken CD8+ T-cell function and induce T-cell exhaustion^17^. PD-L1 and PD-L2, two ligands of PD-1, and their interaction with PD-1 are correlated with T cell activation and tolerance^18^,^19^. However, no literature has reported the possible role of these immune checkpoints in the inflammatory challenge in patients with affective disorders hitherto, and their relationship with known blood level change of cytokines.

This preliminary study investigated the plasma levels of cytokines, proportion of T lymphocytes, and the expression of immune checkpoint inhibitors, including TIM-3, PD-1, PD-L1 and PD-L2, on T lymphocytes and monocytes, in patients with affective disorders.

## Results

### Demographic and clinical characteristics

As shown in Table 1, no significant difference was found in age, gender, marriage, education year, and handedness *(P* > 0.05). However, the age of onset was significantly smaller in patients with BD patients than in patients with MD (22.43 ± 4.50 vs. 29.95 ± 7.79, *P* < 0.001). No significant difference was found in the total course of illness, number of episodes, and total scores of HDRS-17, MADRS and YMRS between these two groups.

### Activation pattern of T lymphocytes and NK cells

**Figure 1:**
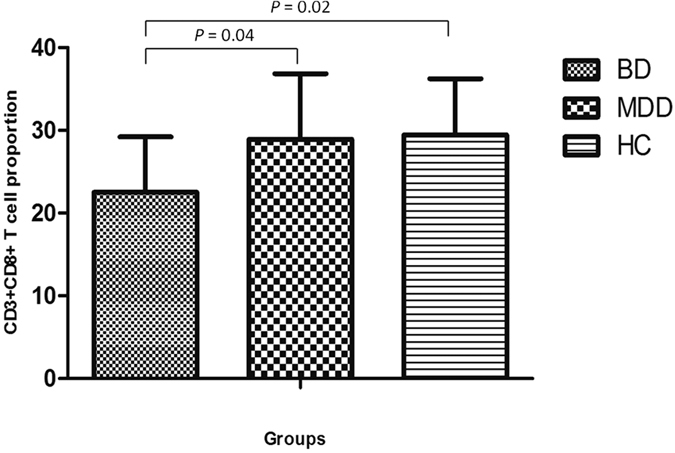
The percentages of peripheral cytotoxic T cells of gated total lymphocytes in patients with BD, MD and healthy controls.

The total blood proportion of CD3+, CD4+, and CD8 + T-cell subsets and CD16 + NK cells was analyzed. No significant difference was found in the percentages of CD3+ and CD4 + T cells and CD16 + NK cells in total lymphocyte populations (Table 2). Of note, the proportion of cytotoxic T cells (CD3 + CD8+) was significantly lower in BD patients than in MD patients and HCs (*P* = 0.004 and, *P* = 0.002, respectively) (Fig. 1).

### Expression of immune checkpoint inhibitors on T lymphocytes and monocytes

**Figure 2:**
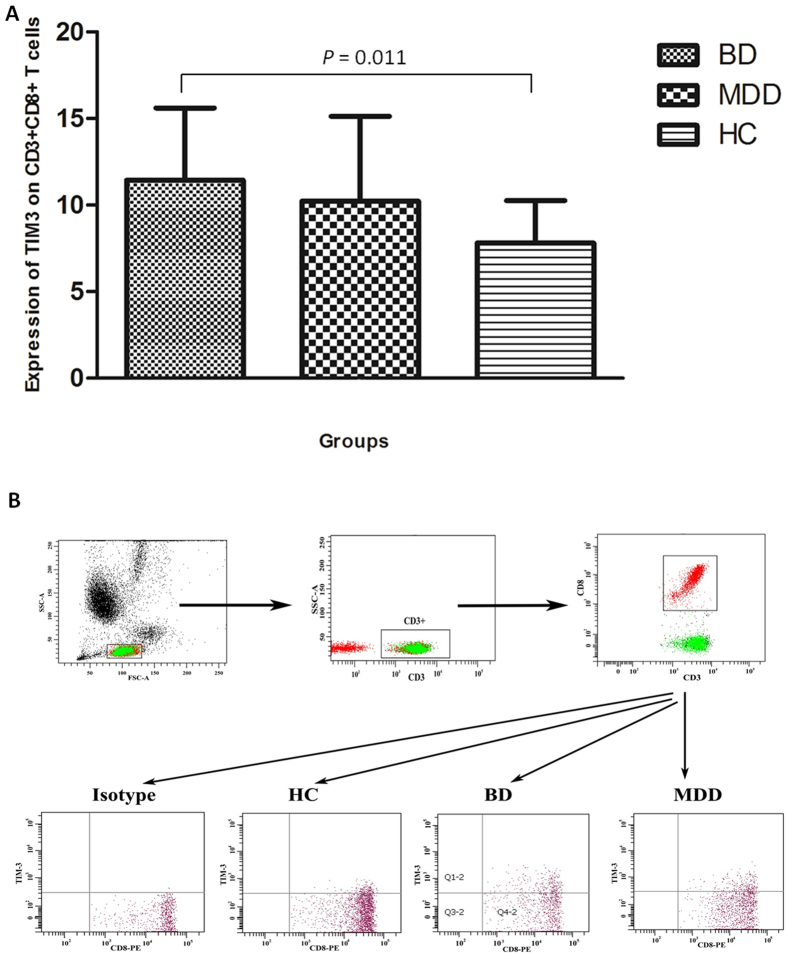
Expression of TIM-3 on cytotoxic (CD3+CD8+) T cells. (**A**) Expression levels of TIM3 on cytotoxic T cells in patients with BD, MD and healthy controls. (**B**) Flow cytometry gating strategy for the determination of TIM-3 (+) on cytotoxic T cells.

**Figure 3:**
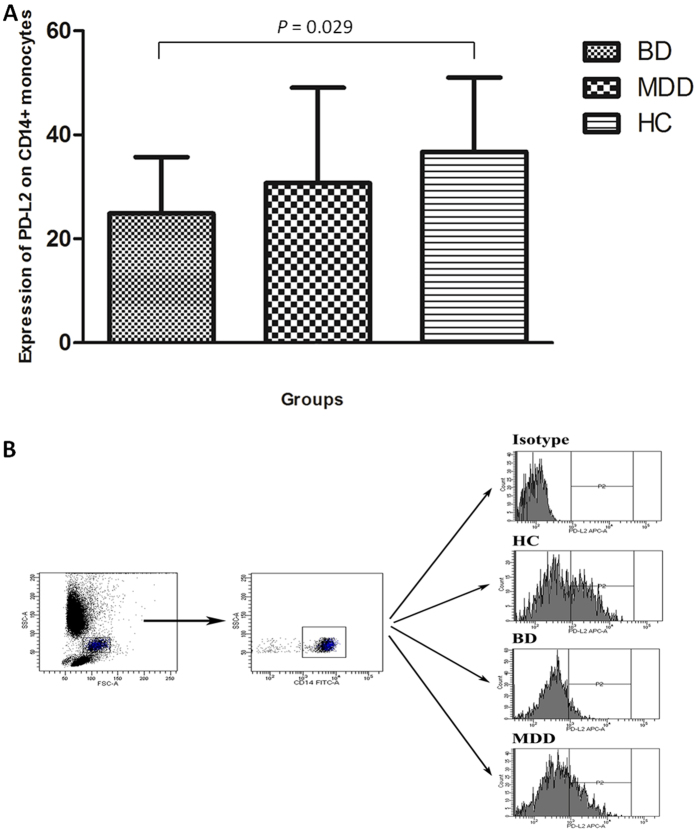
Expression of PD-L2 on CD14+ monocytes. (**A**) Expression levels of PD-L2 on CD14+ monocytes in patients with BD, MD and healthy controls. (**B**) Flow cytometry gating strategy for the determination of PD-L2 (+) on CD14+ monocytes.

In terms of the expression of TIM-3 and PD-1 expression on T-cell subsets, no significant alterations were indicated in the total expression of TIM-3 and PD-1 on T cells (CD3+) or helper T cells (CD3 + CD4+). However, the expression of TIM-3 on cytotoxic T cells (CD3 + CD8+) significantly increased (Fig. 2 and Table 3), but no significant change was revealed in the expression of PD-1. As for CD14+ monocytes, although no significant changes were revealed in the expression of TIM-3, PD-1, and PD-L1 in the three groups, the expression of PD-L2 significantly reduced in patients with BD compared with HCs (24.90 ± 10.84 *vs.* 36.74 ± 14.29, *P* = 0.029, Fig. 3 and Table 3), but not in patients with MD.

### Plasma level of cytokines

**Figure 4:**
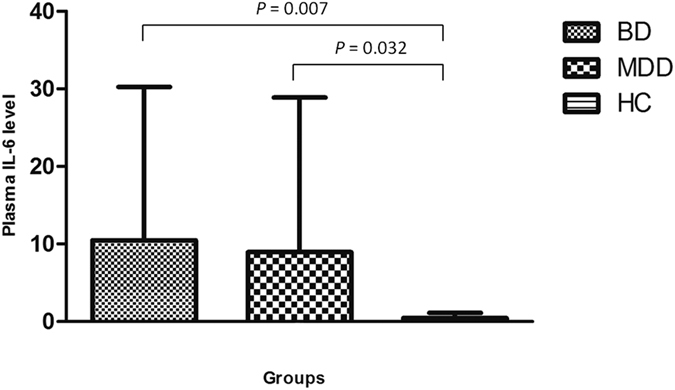
Plasma levels of IL-6 in patients with BD, MD and healthy controls.

Of the cytokines tested, when compared with HCs, the plasma level of IL-6 was significantly elevated in both patients with BD (10.47 ± 19.79 *vs.* 0.44 ± 0.68, *P* = 0.007, Fig. 4) and patients with MD (8.94 ± 19.93 *vs.* 0.44 ± 0.68, *P* = 0.032, Fig. 4). Of note, levels of IL-2, IL-4, IL-10, TNF-α, and IFN-γ in most participants were lower than the set limit of detection, and no significant difference was found among the three groups.

### Correlation analyses

Correlations of immune indices with clinical parameters (onset age, total course of illness, number of episodes, and scores of HDRS-17, MADRS and YMRS) were further examined. In BD patients, the blood proportion of CD3+CD8+ T cells was negatively correlated with the age of onset (*P* = 0.034 and ρ = −0.444). Besides, the expression of TIM-3 on CD3+CD8+ T cells tended to negatively correlate with the MADRS score (*P* = 0.075 and ρ = −0.457). No statistically significant correlations were demonstrated in MD patients.

## Discussion

The present study showed a decreased level of cytotoxic T cells (CD3+CD8+) in patients with BD, accompanied by an elevated concentration of plasma IL-6. Besides, upregulated expression of TIM3 on cytotoxic T cells and downregulated expression of PD-L2 on monocytes were observed in patients with BD, but not in patients with MD. These findings support and extend the understanding of the inflammatory burden in patients with affective disorders, indicating the involvement of immune checkpoint inhibitors in the pathogenesis of BD. In this respect, this work was of importance in extending the research focus from foregone cytokines to immune checkpoints.

In depressive episodes, patients with BD and MD both had an elevated plasma level of IL-6. This was in accordance with the previous evidence the changes of proinflammatory cytokines in patients with affective disorders^20^. As a proinflammatory cytokine, IL-6 was hypothesized to drive the development of depression by impairing serotonin production, enhancing monoamine reuptake, activating the hypothalamic–pituitary–adrenocortical axis^21^. In a STAT3–dependent pattern, IL-6 can directly affect the function of serotonin transporter and consequently induces depression-like behaviour^22^. Moreover, IL-6 and its downstream JAK/STAT pathway participate in facilitating cognitive flexibility^23^. Increased level of IL-6 was also associated with impaired function in reward circuit and other brain regions involved in mood regulation^24^,^25^. However, the exploration of cytokine network is far from enough to uncover the inflammatory changes in patients with MD and BD.

With regard to the T lymphocyte subsets, the proportion of cytotoxic T cells (CD3+CD8+) was reduced in patients with BD, which was negatively associated with the age of onset. These findings indicated that patients with lower number of cytotoxic T cells possibly had an older onset age. In a recent study, a decreased proportion of cytotoxic T cells was also observed in patients with BD^13^, while other studies reported no significant changes^10^,^26^. The primary role of cytotoxic T cells is to mediate cellular immunity against foreign pathogens. A decreased proportion of peripheral cytotoxic T cells might be related to weakened immune defense and increased risk of infection. To date, no research has ever documented whether an increased risk of infection exists in BD patients and its correlation with suppressed activation of cytotoxic T cells. Interestingly, this study was the first to reveal that the expression of TIM-3 on cytotoxic T cells significantly increased in patients with BD, which tented to negatively associate with the score of MADRS. As documented, TIM-3 not only plays a crucial role in mediating the exhaustion of cytotoxic T cells^14^,^17^, but also presents as a potent suppressor of cytotoxic T cell inflammation^27^. Blockade of TIM-3 pathway can help to restore the adaptive immunity^28^. For BD patients, therefore, we speculate that an elevated expression of TIM-3 can lead to decreased number of cytotoxic T cells, suppressed secretion of proinflammatory cytokines from cytotoxic T cells, and eventually play a protective role against the great inflammatory burden during depressive episodes. In this regard, BP patients with reduced number of cytotoxic T cells had a lower overall inflammatory level, which may help to delay the onset of disease.

In addition, the expression of PD-L2 on CD14+ monocytes was downregulated in patients with BD. As another ligand of PD-1, the expression of PD-L2 was more restricted and less extensive than the expression of PD-L1. Similar to PD-L1, engagement PD-1 by PD-L2 was correlated to the downregulation of T-cell proliferation and cytokine production^19^, while knockdown of both PD-L1 and PD-L2 can improve the proliferation and cytokine production of CD4+ T cells^29^. Therefore, the downregulation of PD-L2 on monocytes was possibly correlated with enhanced CD4+ T-cell function, which in turn promoted the production and secretion of proinflammatory cytokines, such as IL-6. However, recent researches have characterized an opposing and competitive role of PD-L1 and PD-L2 in modulating T cell functions during certain pathologic processes, such as allergic asthma^30^. Meanwhile, the essential role of PD-L2 in establishing CD4+ T cell immunity by interfering the PD-1-PD-L1 axis has also been reported^31^. Hence, although the effect of reduced expression of PD-L2 on CD14+ monocytes in patients with BD remains unknown, it will inevitably disrupt the cellular interaction between monocytes and T cells, and as a consequence, cause disturbance in T cell homeostasis. The aforementioned findings would be critical if they are further verified in patients with BD.

The present study had several limitations. One major limitation was the relatively small sample size, which inevitably weakened the statistical power. Another is the detection limit of cytokine level in this study, which appeared to be too high for most cytokines explored and made the statistical results of the cytokine levels less persuasive. In the present study, the role of altered expression of TIM-3 and PD-L2 was not further investigated, which hindered the comprehensive interpretation of our findings.

Above all, the present study favored the immune dysfunction in patients with affective disorders, and called for more attention to be paid to the role of immune checkpoint inhibitors in future studies.

## Methods

### Participants

Between January 2015 and December 2015, 23 patients with BD with depressive episode and 22 patients with major depression were enrolled from inpatient or outpatient sources of the First Affiliated Hospital, Zhejiang University School of Medicine. All participants were in compliance with the following criteria: patients of both sexes were aged from 15 to 53 years; diagnosis was made according to *Diagnostic and Statistical Manual of Mental Disorders*, 4th edition (DSM-IV) criteria and further confirmed by the Structured Clinical Interview for DSM-IV; and the patients scored more than 20 points on the 17-item Hamilton Depression Rating Scale (HDRS-17)^32^. All patients either were drug-naive or discontinued medication for at least 3 months. Besides, 20 age- and gender-matched healthy controls (HCs) without any severe medical condition, major psychiatric disorder, or family history of similar diseases were recruited from local communities. Demographic and clinical variables, including gender, age, marriage, education year, and handedness of all participants, and age of onset, total course of illness, and number of episodes for patients, are presented in Table 1. In addition, the severity of depressive or manic symptoms of patients was characterized by the HDRS-17, the Montgomery Asberg Depression Rating Scale (MADRS)^33^, and the Young Mania Rating Scale (YMRS)^34^. Exclusion criteria for both patients and controls included: (a) history of other psychiatric or neurological disorders, or other significant somatic diseases (including hypertension, hyperlipidemia, diabetes mellitus, and other chronic diseases); (b) history of brain injury; (c) history of suicidal attempts; (d) history of acute infection 3 months before commencement of the study; (e) history of pregnancy or lactation 6 months before commencement of the study; and (f) use of immune-regulatory drugs or alcohol/drug dependence history 6 months before commencement of the study.

This study was approved by the Hospital Ethical Committee of the First Affiliated Hospital, Zhejiang University School of Medicine, and was performed in accordance with the principles of the Declaration of Helsinki. All participants, or their guardians, signed informed consent before enrollment.

### Measurement of cell surface markers

Venous blood samples (1 mL) were collected from individual patients and healthy individuals immediately after visiting the hospital. To determine the frequency of T cells (CD3/CD4/CD8), natural killer (NK) cells, and monocytes (CD14), blood samples were stained with the following antibodies: Pcy5-conjugated anti-CD3, FITC-conjugated anti-CD4, P-phycoerythrin-conjugated anti-CD8 (BD Biosciences, CA, USA), and PE-conjugated anti-CD16/CD56 (Beckman Coulter, CA, USA).

The frequency of different types of immunocompetent cells was characterized by flow cytometry as previously described (PMID: 8404602). Briefly, at least 1 × 10^5^ cells were analyzed using BD FACS Canto^TM^ II flow cytometer (BD Bioscience, CA, USA). The staining of T cells or monocytes was performed with a single antibody or a combination of the following antibodies: anti-Tim-3-APC (R&D Systems), anti-PD-1-PE (eBiosciences), anti-PD-L1-BrilliantViolet421 (Biolegend), and anti-PD-L2-APC (Biolegend). The isotype-matched immunoglobulins were used as controls. The tests were performed at least in triplicate.

### Plasma cytokine determination

In the present study, the levels of plasma cytokines, including IFN-γ, TNF-α, IL-2, IL-4, IL-6, and IL-10, were also determined using BD cytometric bead array human Th1/Th2 cytokine kit (BD Bioscience), according to the manufacturer instructions. The concentrations of plasma cytokines were determined using standard curves established with the individual recombinant cytokines provided. The limitation of detection was 7.1 pg/mL for IFN-γ, 2.8 pg/mL for TNF-α, 2.6 pg/mL for IL-2, 2.6 pg/mL for IL-4, 3.0 pg/mL for IL-6, and 2.8 pg/mL for IL-10. All these tests were carried out in triplicate.

### Statistical interpretation

Statistical analysis was conducted using the SPSS 17.0 statistical software (IBM, IL, USA). When data were not qualified for normal distribution even after transformation, they were analyzed using Kruskal–Wallis test, or otherwise, one-way analysis of variance and *t* test were used. The difference between proportions was analyzed using the chi-square test. Correlation analyses between the expression of cell surface markers, plasma cytokines and clinical parameters were performed by Spearman’s rho test. The significance level was set at two-tailed *P* value < 0.05.

## Additional Information

**How to cite this article**: Wu, W. *et al*. Circulating T lymphocyte subsets, cytokines, and immune checkpoint inhibitors in patients with bipolar II or major depression: a preliminary study. *Sci. Rep.* **7**, 40530; doi: 10.1038/srep40530 (2017).

**Publisher's note:** Springer Nature remains neutral with regard to jurisdictional claims in published maps and institutional affiliations.

## Figures and Tables

**Table 1:** Demographic and clinical characteristics of the study subjects (mean ± SD).

	BD (n = 23)	MD (n = 22)	HC (n = 20)	P
Age	31.61 ± 9.93	35.68 ± 11.14	35.75 ± 7.43	0.271^†^
Sex, Female, %	47.83	54.55	45	0.815^#^
Marriage, Married, %	56.52	68.18	75	0.429^#^
Education (>12 years) %	39.13	50	45	0.287^#^
Right hand, %	100%	100%	100%	1^#^
Onset age	22.43 ± 4.50	29.95 ± 7.79	—	<0.001^*△^
Duration of illness (year)	8.52 ± 6.63	5.82 ± 3.89	—	0.104^△^
Number of episodes	5.52 ± 3.16	3.95 ± 2.46	—	0.071^△^
HDRS score	25.57 ± 5.96	25.73 ± 7.25	—	0.935^△^
MADRS score	25.91 ± 3.01	23.91 ± 6.31	—	0.178^△^
YMRS scores	3.39 ± 2.04	3.59 ± 1.47	—	0.709^△^

^*^P < 0.05 (two-tailed). Abbreviations: BD, bipolar disorder; MD, major depression; HC, healthy control; HDRS, Hamilton Depression Rating Scale; MADRS, Montgomery Asberg Depression Rating Scale; YMRS, Young Mania Rating Scale; SD: standard deviation.

^†^One-way ANOVA test; ^#^Pearson chi-square test; ^Δ^Independent samples t test.

**Table 2:** Lymphocytes subsets presented as percentages of total lymphocytes gated (mean ± SD).

Cell Type	BD (n = 23)	MD (n = 22)	HC (n = 20)	P1	P2	P3	P4
CD3+ T cell	66.59 ± 10.70	68.56 ± 9.98	67.20 ± 9.41	0.844	0.663	0.514	0.800
CD3+CD4+ Th cell	36.07 ± 8.00	35.40 ± 8.05	33.51 ± 7.53	0.292	0.440	0.777	0.554
CD3+CD8+ Tc cell	22.50 ± 6.73	28.93 ± 7.93	29.45 ± 6.80	0.002*	0.816	0.004*	0.003*
CD3-CD16+CD56+NK cell	18.88 ± 8.67	18.34 ± 9.85	21.59 ± 8.18	0.368	0.286	0.841	0.534

^*^P < 0.05 (two-tailed). Abbreviations: Th, helper T cell; Tc, T cytotoxic cell; NK, Natural Killer cell.

*P*_1_: BD and HC; *P*_2_: MD and HC; *P*_3_: BD and MD; *P*_4_: BD, MD and HC.

**Table 3:** Levels of immune checkpoint inhibitors on immune cells in patients with BD, MD and controls (mean ± SD).

Immune checkpoint	BD (n = 23)	MD (n = 22)	HC (n = 20)	*P*_*1*_	*P*_*2*_	*P*_*3*_	*P*_*4*_
T cell total TIM-3^†^	6.43 ± 2.62	7.91 ± 4.63	6.55 ± 2.20	0.920	0.243	0.219	0.378
T cell total PD-1^†^	16.6 ± 4.55	22.55 ± 8.74	16.65 ± 4.94	0.985	0.072	0.083	0.160
CD4+TIM-3+^△^	3.68 ± 2.12	3.61 ± 1.94	3.08 ± 1.04	0.681	0.668	0.856	0.882
CD4+PD-1+^†^	19.70 ± 9.70	19.50 ± 12.01	16.17 ± 5.55	0.343	0.459	0.966	0.573
CD8+TIM-3+^†^	11.45 ± 4.15	10.22 ± 4.90	7.81 ± 2.44	0.011^*^	0.082	0.379	0.034^*^
CD8+PD-1+^†^	16.19 ± 5.30	19.10 ± 11.59	19.03 ± 6.80	0.385	0.986	0.488	0.644
CD14+TIM-3+^†^	84.42 ± 19.75	81.69 ± 15.68	83.85 ± 11.41	0.920	0.692	0.648	0.885
CD14+PD-1+^△^	9.78 ± 8.97	4.72 ± 3.02	7.24 ± 4.14	1.000	0.206	0.350	0.465
CD14+PD-L1+^△^	5.51 ± 5.86	7.89 ± 9.33	3.56 ± 2.49	0.913	0.079	0.318	0.257
CD14+PD-L2+^†^	24.90 ± 10.84	30.74 ± 18.33	36.74 ± 14.29	0.029^*^	0.242	0.291	0.088

^*^P < 0.05 (two-tailed). *P*_1_: BD and HC; *P*_2_: MD and HC; *P*_3_: BD and MD; *P*_4_: BD, MD and HC.

^†^One-way ANOVA test; ^Δ^Kruskall–Wallis test.
